# The Effectiveness of Plasma Rich in Growth Factors (PRGF) in the Treatment of Nerve Compression Syndromes of the Upper Extremity: A Retrospective Observational Clinical Study

**DOI:** 10.3390/jcm11164789

**Published:** 2022-08-16

**Authors:** Víctor Galán, Iñaki Iñigo-Dendariarena, Iñigo Galán, Roberto Prado, Sabino Padilla, Eduardo Anitua

**Affiliations:** 1 Hand, Wrist and Microsurgery Unit, Clínica Indautxu, 48010 Bilbao, Spain; 2 School of Medicine, European University, 28670 Madrid, Spain; 3 BTI-Biotechnology Institute I MAS D, 01007 Vitoria, Spain; 4 University Institute for Regenerative Medicine and Oral Implantology-UIRMI (UPV/EHU-Fundación Eduardo Anitua), 01007 Vitoria, Spain; 5 Eduardo Anitua Foundation for Biomedical Research, 01007 Vitoria, Spain

**Keywords:** pain, platelet-rich plasma, PRGF, nerve compression syndrome, carpal tunnel syndrome, ulnar nerve entrapment, intraneural injection, regenerative medicine

## Abstract

Background: Nerve compression syndromes of the upper extremity are a common cause of neuropathic pain and functional impairment. Recently, platelet-rich plasma (PRP) infiltrations have emerged as an effective biological approach to the treatment of this type of injury. The objectives of this retrospective observational study were to assess clinical improvement in patients with median and ulnar nerve entrapment syndrome after undergoing biologically-assisted nerve release surgery with plasma-rich-in-growth-factors (PRGF) technology. Methods: Participants (*n* = 39) with moderate-to-severe nerve compression syndrome of the upper limb diagnosed by both electromyography and clinical examination, and who were treated with PRGF, were identified from the center’s medical records. The evaluation was based on patient-reported outcomes. Pre- and post-treatment differences in the Visual analog scale (VAS), the Boston carpal tunnel questionnaire (BCTQ), and the Quick-DASH score were assessed. Results: Three study groups were conducted: patients with carpal tunnel syndrome (*n* = 16), with recurrent carpal tunnel syndrome (*n* = 8), and with ulnar nerve entrapment (*n* = 15). The median follow-up was 12 months (interquartile range (IQR), 9–16). In comparison to pre-treatment values, all three study groups obtained statistically significant improvements for the three analyzed scales at the end of the follow-up, with *p* < 0.001 for all scales in the carpal tunnel syndrome and ulnar nerve entrapment groups and *p* < 0.01 for all scales in the recurrent carpal tunnel syndrome group. There were no serious adverse effects in the analyzed patients. Conclusion: PRGF-assisted open surgical nerve release treatment (intraneural and perineural liquid PRGF infiltrations and nerve wrapping with PRGF membrane) exerts long-term beneficial effects on pain reduction and functional improvement in the nerve and nerve–muscle unit in patients with upper extremity compression syndromes.

## 1. Introduction

Nerve compression syndromes of the upper extremity, including ulnar nerve entrapment (UNE) and carpal tunnel syndrome (CTS), are a common cause of elbow, hand, finger, and wrist neuropathic pain and functional impairment [1]. In conservative and surgical treatment of peripheral nerve entrapment syndromes, numerous options have been applied with disparate results [2,3]. For instance, after 1–3 years of conservative treatment, almost 65% of patients with CTS underwent surgical nerve release from the ligament entrapment [4,5]. However, surgical intervention does not influence the chronic intraneural fibrosis that the entrapped nerve undergoes, with the failure rate of surgery alone ranging from 7 to 75% [3]. In recent years, platelet-rich plasma (PRP) infiltrations have emerged as an efficacious molecular intervention in patients with mild-to-moderate CTS or as adjuvant therapy to assist surgically repaired damaged nerves including the radial, ulnar, and median [3,4,6,7,8,9,10,11]. Different types of PRP—depending on their platelet concentrations, activation processes, and the presence or absence of leukocytes including leukocyte-rich plasma (L-PRP), platelet-rich fibrin (PRF) or plasma rich in growth factors (PRGF)—have emerged in the last few years for the treatment of neuropathies [12,13]. PRP has been applied as a liquid-to-gel product intraneurally and perineurally and/or by wrapping the repaired nerve area with a PRP matrix–like viscous and malleable membrane [7,8,9,10,11]. Considerable evidence in both preclinical and clinical settings indicates that PRP products act as a molecular intervention with neurotrophic (NGF, BDNF, IGF-1, PDGF, VEGF, and HGF) and neurotropic (fibrin, fibronectin, and vitronectin) factors [9,14]. These growth factors (GFs), microparticles, and other bioactive mediators are instrumental agents that stimulate angiogenesis; exert antiapoptotic, antifibrotic, and neuroprotective effects on injured neural tissues; dampen early inflammation; accelerate spontaneous axonal growth; attenuate muscle atrophy; and modulate stem cell–like myelinating Schwann cell activation and macrophage polarization, thereby acting as key drivers of full nerve function recovery [7,9,11,15].

Our study aimed at the appraisal of clinical improvement in patients with median and ulnar nerve entrapment syndrome six months to two years after undergoing nerve release surgery assisted by PRGF intraneural and perineural infiltrations and PRGF membranes by using the Visual analog scale (VAS), the Boston carpal tunnel questionnaire (BCTQ), and the Quick-DASH score.

To the best of our knowledge, this novel combination (intraneural infiltration, perineural infiltration, and fibrin membrane around the injured nerve) has only been described previously by Sanchez et al. in a sheep regeneration model and in a description of the technique [16,17].

## 2. Materials and Methods

### 2.1. Study Design and Data Source

This retrospective study was reported following the Strengthening the Reporting of Observational Studies in Epidemiology (STROBE) statement guidelines (Supplementary Table S1) [18]. The study protocol (VG-01-ER-22) was approved on 15 February 2022 by the Institutional Review Board (CEIm-E) in accordance with the international ethical standards from the revised World Medical Association Declaration of Helsinki amended in 2013 in Brazil [19].

We retrospectively reviewed the anonymized database of patients with nerve compression syndromes of the upper extremity treated with PRGF infiltrations in the Hand, Wrist and Microsurgery Unit of the Indautxu Clinic between January 2017 and December 2021.

The inclusion criteria were the following: (1) patients of both sexes over 18 years old (2) with moderate-to-severe nerve compression syndrome of the upper limb diagnosed by electromyogram and clinical examination (3) who have been treated with PRGF, (4) have completed at least one evaluation scale of the upper limb (prior to the intervention with PRGF and at the end of their follow-up), and (5) with a minimum follow-up of six months. The exclusion criterion was lack of complete records of the variables to be studied.

### 2.2. PRGF Preparation

PRP was elaborated according to PRGF-Endoret technology (BTI Biotechnology Institute, Vitoria, Spain). Briefly, a total of 36 mL of peripheral venous blood was withdrawn into 9-mL tubes containing 3.8% (*w*/*v*) sodium citrate. The blood was centrifuged at 580 g for 8 min at room temperature. The upper volume of the plasma, which contains a similar platelet count to that of peripheral blood (F1), was drawn off and kept in a collection tube to prepare the membrane. The 2-mL plasma fraction (F2), located just above the sediment red blood cells but not including the buffy coat, was collected in another tube and carried to the injection room ready for use. This F2 plasma contained a moderate enrichment of platelets (approximately 2-fold the platelet count of the peripheral blood) with scarce leucocytes. To initiate clotting and platelet activation, calcium chloride (10 % *w*/*v*) was added to the liquid F2 just before injection. In order to generate a PRGF membrane, 6–8 mL of the PRGF F1 was activated with calcium chloride (10% *w*/*v*) and incubated at 37 °C for 20–30 min in a glass dish, to allow the formation of a biocompatible fibrin membrane which is then wrapped around the freed-up nerve portion. All the procedures were performed under sterile conditions in the operating room.

PRGF contains neither leukocytes nor erythrocytes and can therefore be classified as pure-PRP (P-PRP), specifically type 4-B [20], P2-x-Bβ category [21], and 24-00-11 [22] according to three classifications that have been proposed for PRP.

### 2.3. Medical Intervention

**Figure 1:**
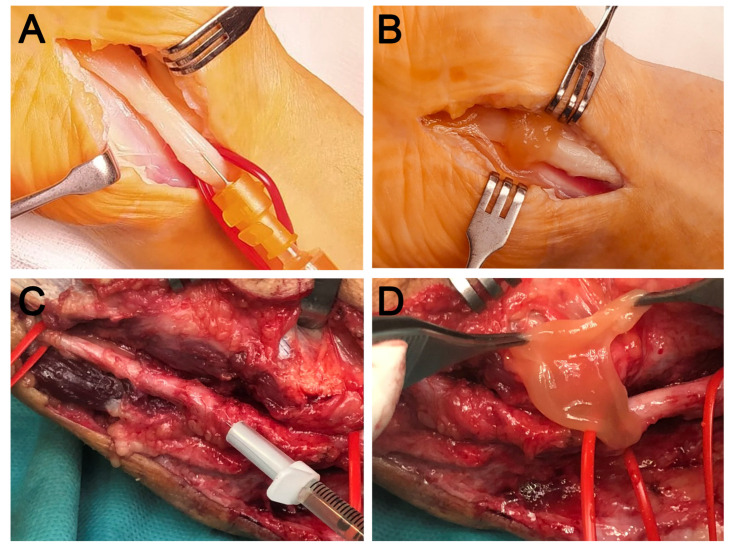
Representative images of two cases of nerve compression in the upper limb. Open surgical release of the carpal retinaculum (**A**,**B**) and of the ulnar epitrochlear ligament (**C**,**D**). In the case of the median nerve, intraneural PRGF (**A**) is applied after release and subsequently wrapped with a PRGF membrane (**B**). Similarly, in the case of the ulnar nerve, intraneural infiltration is performed after neurolysis (**C**), followed by the application of a PRGF fibrin membrane (**D**) in order to avoid postoperative fibrotic entrapment.

Open surgical release of the carpal retinaculum or ulnar epitroclear ligament was performed by the same experienced surgeon. Once the entrapped nerve was released, the surgeon proceeded to apply PRGF. Firstly, 2 to 4 mL of the F2 PRGF in its liquid formulation was, once activated, injected gently with slow speed using a 22 G needle intraneurally and perineurally in the entrapped area as well as proximally and distally to the entrapped nerve area. Secondly, the affected area of the nerve around the ligament was wrapped with a matrix-like viscous and malleable membrane of the F1 PRGF which acts as a transient guiding conduct, thereby preventing post-surgical fibrotic entrapment [16,23]. Figure 1 shows representative images of the procedure for both the median and ulnar nerves.

### 2.4. Outcome Measurements

Evaluation of the results was carried out by means of patient-reported outcomes. Specifically, three different scores were carried out to assess pain and nerve functional impairment as a baseline 6 to 24 months after the surgical and molecular intervention: Visual analog scale (VAS), Boston carpal tunnel questionnaire (BCTQ), and Quick-DASH score.

BCTQ comprises two distinct scales: the Symptom Severity Scale (SSS), which has eleven questions assessing pain, paresthesia, numbness, weakness, nocturnal symptoms, and difficulty of grasping and uses a five-point rating scale; and the Functional Status Scale (FSS), which has eight questions assessing the degree of difficulty (also on a five-point rating scale) in several activities (writing, buttoning clothes, holding a book while reading, gripping a telephone handle, opening jars, performing household chores, carrying grocery bags, bathing, and dressing). Each subscale generates a final score. A higher score indicates greater disability [24].

The Quick-DASH score allows the evaluation of the inability perceived by the patient to perform certain activities. It consists of 11 questions; the first 6 measure the degree of difficulty in performing several physical activities, and the other 5 are related to quality of sleeping, social activities, daily activities, and the intensity of pain and numbness. All the questions are scored on a scale of 5 levels (from 1 to 5). A higher score indicates greater disability [25].

Data were obtained from the three scales for patients with median nerve entrapment syndrome, while for patients with ulnar entrapment, data were obtained only from the Quick-DASH score.

In addition, adverse event data were obtained from the medical records of every patient.

### 2.5. Statistical Analysis

Descriptive data were presented as frequencies and percentages. The results of the questionnaires were presented as median [interquartile ranges]. All the data values were tested for normality using the Shapiro–Wilk test. We display box-and-whisker plots following the Tukey style: the boxes show the median and the interquartile range (IQR), while the whiskers indicate the 25th percentile −1.5 × the IQR and the 75th percentile −1.5 × IQR. The square points indicate outliers outside the whisker range [26]. Changes in outcome measures between pre- and post-treatment were assessed using the Wilcoxon signed-rank test. Differences were considered statistically significant for a value of *p* < 0.05. The statistical analyses were performed with SPSS software (version 15.0; IBM Corp., Chicago, IL, USA).

## 3. Results

This study included 39 patients with nerve compression syndromes of the upper extremity, including 24 with carpal tunnel syndrome and 15 with ulnar nerve entrapment. Eight of the 24 patients with carpal tunnel syndrome had undergone one previous surgery (without PRGF) and presented recurrent CTS. The baseline demographic features of the patients are shown in Table 1. All the patients with CTS presented positive Phalen’s test and/or Tinel’s sign. Three study groups were formed on the basis of their distinctive characteristics: on the one hand, depending on the type of nerve affected (median or ulnar), and on the other hand, depending on whether the CTS had been present for the first time or was recurrent to a first surgical intervention (difficult-to-treat condition), since the clinical implications are different in each case.

### 3.1. Carpal Tunnel Syndrome Group (CTS)

**Figure 2:**
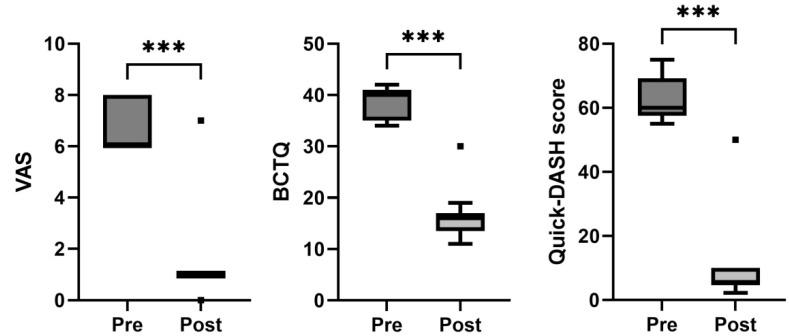
Box-and-whisker plots showing the results of the carpal tunnel syndrome for visual analogue scale (VAS), Boston Carpal Tunnel Questionnaire (BCTQ), and Quick-DASH score. The boxes show the median and interquartile range (IQR), and the whiskers indicate the 25th percentile −1.5 × IQR, and the 75th percentile −1.5 × IQR. The square points indicate outliers outside the whisker range. *** indicates *p* < 0.001 (*n* = 16).

Comparing the baseline data, a statistically significant improvement in the VAS (*p* < 0.001), BCTQ (*p* < 0.001), and Quick-DASH scores (*p* < 0.001) was observed at the follow-up assessment for this group of patients (Figure 2 and Table 2). Only one patient out of sixteen maintained a positive Phalen’s test and Tinel’s sign after the follow-up period.

### 3.2. Recurrent Carpal Tunnel Syndrome Group (RCTS)

**Figure 3:**
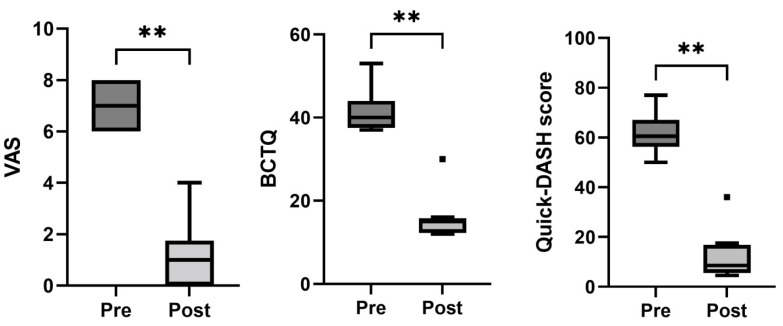
Box-and-whisker plots showing the results of the recurrent carpal tunnel syndrome group for visual analogue scale (VAS), Boston Carpal Tunnel Questionnaire (BCTQ), and Quick-DASH score. The boxes show the median and interquartile range (IQR), and the whiskers indicate the 25th percentile −1.5 × the IQR and the 75th percentile −1.5 × IQR. The square points indicate outliers outside the whisker range. ** indicates *p* < 0.01 (*n* = 8).

All the patients in this group had undergone a first surgery before our surgical intervention assisted by PRGF. Compared with the baseline data, a statistically significant improvement in the VAS (*p* < 0.01), BCTQ (*p* < 0.01), and Quick-DASH scores (*p* < 0.01) was observed at the end of the follow-up. (Table 2 and Figure 3). In this case, five patients maintained a positive Phalen’s test, while three had a positive Tinel’s sign after the follow-up period.

### 3.3. Entrapment of the Ulnar Nerve Group (EUN)

In this group of patients, we only carried out the Quick-DASH score. We observed a statistically significant improvement comparing the baseline outcome after the intervention (*p* < 0.001) (Table 2).

### 3.4. Adverse Events

With respect to adverse effects, PRGF treatment did not cause any serious adverse effects in the analyzed patients.

## 4. Discussion

This retrospective observational clinical study confirms that a combinatorial approach to treat peripheral nerve entrapment—namely, open surgical nerve release assisted by intraneural and perineural PRGF liquid and membrane applications—has a long-term positive impact on pain reduction and functional amelioration of nerve and nerve–muscle unit function in patients with ulnar and median compression syndrome. These results are consistent with previous studies applying PRP alone or in combination with open surgical release of the entrapped nerve assisted by PRP infiltration around the nerve [3,4,6,8,15,27]. Recently, a prospective clinical trial on mild-to-extreme CTS showed that performing open surgical release of the carpal ligament combined with 3 mL of PRP infiltration around the median nerve led to clinical and functional improvements 6 weeks after the procedure [8]. Similar results were obtained using only a single ultrasound-guided PRP injection to peel the median off the retinaculum via hydrodissection as a treatment to tackle mild-to-moderate CTS [3]. Wu et al. [3] demonstrated that this modality of treatment reduced the pain level (VAS scale) from 6.5 ± 0.30 to 2.91 ± 0.23 and 1.97 ± 0.23 at three and six months post-injection, results comparable with the pain reductions shown in our study with an average follow-up of 10.9 ± 3.5 months (from 6.9 ± 1 to 1.3 ± 1.5). Moreover, severity and functional improvement assessed by the BCTQ test results after six months post–PRP procedure presented by Wu et al. [3] (14.1 ±.0.45 and 10.41 ± 0.48) are very close to the values of our study for the CTS and RCTS groups, respectively, but with a longer-term follow-up.

Biological therapies with blood derivatives, such as PRGF, have been spreading to all areas of medicine. However, this type of therapy is in its infancy and still needs to establish an optimal formulation for each pathology and patient (personalized medicine). Nowadays there are many formulations, or products, which vary in the way they are obtained, thus creating variability in their components, e.g., platelets, erythrocytes, and leukocytes [28]. Thus, different products can give rise to different clinical outcomes. Part of the controversy in the use of PRPs lies in their different composition [22], a controversy fueled by the lack of methodological information in many studies [29]. The administration protocol, the pathology, and the patient him- or herself are the other factors responsible for the efficacy of PRP [30].

The idea of applying a combination of nerve release surgery assisted by PRGF intraneural and perineural infiltrations and PRGF membranes stems from the fact that a nerve chronically entrapped undergoes perineural and intraneural fibrosis, which can only be avoided and solved by means of a surgical nerve release assisted by an antifibrotic molecular intervention [9,10,23]. Moreover, by infiltrating PRGF intraneurally and perineurally, and wrapping PRGF membrane as a matrix-like viscous and malleable structure around the injured nerve gap, tissue fibrinolysis breaks the fibrin down, thereby releasing cell-signaling molecules including but not limited to nerve growth factor NGF, BDNF, IGF-1, PDGF, VEGF, HGF, TGF-β, fibronectin, and vitronectin. This retrospective observational clinical study shows that the approach proposed by Sanchez et al. [9] tackles both drawbacks presented by chronic compression syndromes of the upper extremity, somehow reverting nerve–muscle unit functionality [17]. Importantly, we included the RCTS group as a difficult-to-treat condition and with no other therapeutic options. Significantly, patients in this RCTS group showed that a second surgical intervention assisted by PRGF significantly ameliorated pain and nerve function assessed by the VAS scale, the BCTQ, and the Quick-DASH score.

The exact molecular mechanisms by which PRP would reduce pain are not yet fully understood. Among them, the reduction in proinflammatory molecules and the resolution of inflammation, the upregulation of endocanabinoid receptors type 1 and 2, and the regeneration of axons and target reinnervation could be responsible [11,13,31,32].

Study Limitations

This study, however, presents several limitations. The first is related to its design as a retrospective study, and not all patients had the same follow-up period, causing the studied follow-up to range from 6 to 24 months. However, an average follow-up time of 12 months as carried out in this study might be considered as a long-term follow-up. Another limitation is the small sample size of the RCTS group compared to the CTS and EUN groups. Nevertheless, and due to the challenging pathology shown by patients with recurrent CTS, we considered it significant to include this group in the study and extend this new treatment to a new difficult-to-treat pathology. Finally, we did not assess the molecular mechanisms underlying this therapeutic approach. Despite that, considerable evidence in basic science and a clinical setting indicates the positive effects of PRPs on peripheral neuropathies. Future studies should explore the influence of different GFs on the neuroprotective, anti-inflammatory, and antifibrotic effects of PRPs on chronically entrapped nerves.

## 5. Conclusions

In conclusion, this study shows that combining open surgical nerve release with intraneural and perineural PRGF liquid and membrane applications exerts long-term beneficial effects on pain reduction and functional amelioration of nerve and nerve–muscle unit function in patients with ulnar and median compression syndrome.

## Figures and Tables

**Table 1:** Demographic characteristics of the patients.

	**Total**	**Subgroups**		
		Carpal Tunnel Syndrome	Recurrent Carpal Tunnel Syndrome	Entrapment of the Ulnar Nerve
Number of patients (*n*)	39	16	8	15
Age (years, mean ± SD)	53.6 ± 11.4	52.6 ± 10.2	60.6 ± 14.8	51.1 ± 9.7
Women (number, %)	23 (59%)	14 (87.5%)	5 (62.5%)	4 (26.7%)
Follow-up (months, median (IQR ^1^))	12 (9–16)	12 (8–14)	10 (6–12)	18 (16–24)
Repetitive work movements (number, %)	21 (53.8%)	10 (62.5%)	4 (50%)	7 (46.7%)

^1^ IQR, interquartile range.

**Table 2:** Comparison of changes in patient-reported outcomes. Results are reported as median [interquartile ranges].

	Groups	Pre-Treatment	Post-Treatment	*p* Value ^1^
Carpal tunnel syndrome (CTS) group (*n* = 16)				
	VAS scale	6 (6–8)	1 (1–1)	<0.001
	Boston Carpal Tunnel Questionnaire	40 (35–41)	16 (13.5–17)	<0.001
	Quick-DASH score	60 (57.5–69.2)	5.65 (4.6–10)	<0.001
Recurrent carpal tunnel syndrome (RCTS) group (*n* = 8)				
	VAS scale	7 (6–8)	1 (0–1.8)	<0.01
	Boston Carpal Tunnel Questionnaire	40 (37.5–44)	15 (12.3–15.8)	<0.01
	Quick-DASH score	60.5 (56.3–67)	8.5 (5.5–16.9)	<0.01
Entrapment of the ulnar nerve (EUN) group (*n* = 15)				
	Quick-DASH score	60 (58–65)	14 (10–16)	<0.001

^1^ Wilcoxon signed-rank test.

## Data Availability

All the obtained data used to support the findings of this study are available from the corresponding author upon reasonable request.

## Supplementary Materials

The following supporting information can be downloaded at: www.mdpi.com/article/10.3390/jcm11164789/s1, Supplementary Table S1: STROBE (Strengthening the Reporting of Observational Studies in Epidemiology) statement checklist.

## Abbreviations

BCTQ	Boston Carpal Tunnel Questionnaire
CTS	carpal tunnel syndrome
GFs	growth factors
PRGF	plasma rich in growth factors
PRP	platelet-rich plasma
UNE	ulnar nerve entrapment
VAS	visual analogue scale
